# Unexpected High Ductility of Fe40Al Alloys at Room Temperature

**DOI:** 10.3390/ma14174906

**Published:** 2021-08-28

**Authors:** Dariusz Siemiaszko, Iwona Garwacka

**Affiliations:** Faculty of Advanced Technologies and Chemistry, Military University of Technology, Gen. Sylwestra Kaliskiego 2, 00-908 Warsaw, Poland; garwacka12@gmail.com

**Keywords:** FeAl alloys, sintering, ductility

## Abstract

Iron aluminium alloys, especially those sintered from elemental powders, suffer from low ductility. In this paper, an iron aluminium alloy (Fe40Al) produced by pressure-assisted induction sintering from elemental powders is shown and described. Samples produced by this method show an unexpectedly high ductility in the compression test that is an order of magnitude higher than the literature values. Microstructural observations show plastic behaviour with significant deformation of the grains and a lack of decohesion. At the same time, the tensile properties of these samples remain at much lower levels. An attempt to explain this phenomenon is made and described in this paper.

## 1. Introduction

Alloys based on intermetallic phases have been intensively studied for several decades. The results of the first studies were very promising and indicated that alloys based on phases from the Fe–Al or Ni–Al system have the potential to replace expensive stainless steels in the industry [1,2]. However, to be practically applicable, in addition to many advantages, such as low density, good corrosion and oxidation resistance, and high strength at room and elevated temperatures, the phases from the Fe–Al system should also be characterised by good or at least reasonable ductility and impact toughness.

The yield point of alloys from the Fe–Al system is relatively low and is usually in the range of 300–500 MPa [3]. As [4] has shown, single crystals of Fe-39 at.% Al stretched in air exhibit a very high elongation of 30%. Moreover, basic research has shown that this phase deforms by <111> slip on the {110} planes, which makes possible the operation of five independent slip systems. Therefore, this material meets the von Mises condition, which specifies the minimum conditions for plastic deformation.

Unfortunately, despite these advantages shown on model materials under precisely controlled conditions, the properties of classically manufactured samples turned out to be completely different. Alloys from the Fe–Al system were found to be brittle, especially at room temperature [2,5]. This brittleness practically prevents plastic from forming and limits its applicability to casted materials. Fe–Al alloys, instead of becoming the material of the “future”, joined the group of materials that failed the hopes placed in them. Their brittleness has at least three causes:

- Environmental brittleness, i.e., the action of water vapour, which, even at a very low pressure of 1.33 × 10^−5^ Pa, reacts with aluminium to form aluminium oxide and hydrogen. Hydrogen penetrates the material, making it brittle [6,7,8,9];
- A low cohesive strength of grain boundaries resulting from long-range order—the greater the degree of order is, the lower the cohesion will be [10,11,12];
- Hardening as a result of the presence of thermal vacancies, which reduces plasticity [13,14,15,16,17].

**Figure 1:**
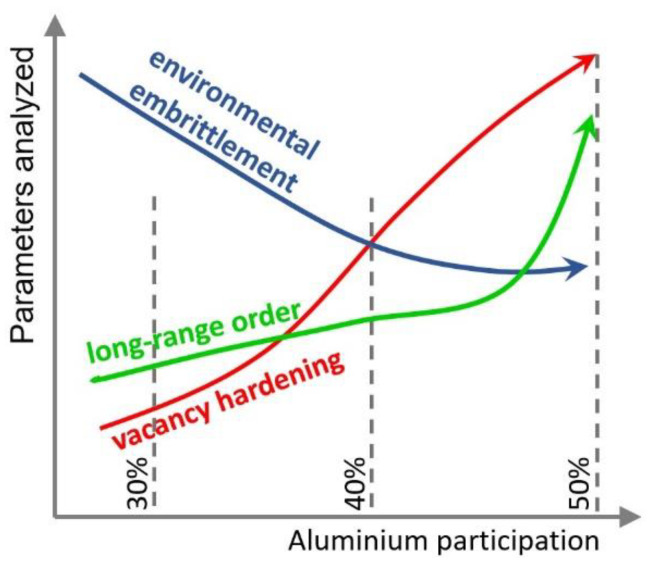
Changes in the factors influencing the brittleness of the FeAl phase depending on the aluminium content. Based on [6,7,8,9,10,11,12,13,14,15,16,17].

The nature of the influence of all three causes strongly depends on the content of aluminium in the alloy. Figure 1 shows changes in individual parameters as a function of aluminium content. It is seen that these changes are not linear. Studies have shown that environmental brittleness decreases with increasing aluminium content to 50%. This behaviour is most likely due to the formation of a protective Al_2_O_3_ layer, which makes contact with water vapour difficult. In alloys with aluminium contents over 40%, the influence of hydrogen embrittlement is insignificant. Unfortunately, instead of falling, the brittleness of these alloys increases even more for two other reasons: the degree of order and the strengthening by vacancies. Hence, the most popular alloys have an aluminium content of approx. 40%, the plastic properties of which seem to be optimal.

**Figure 2:**
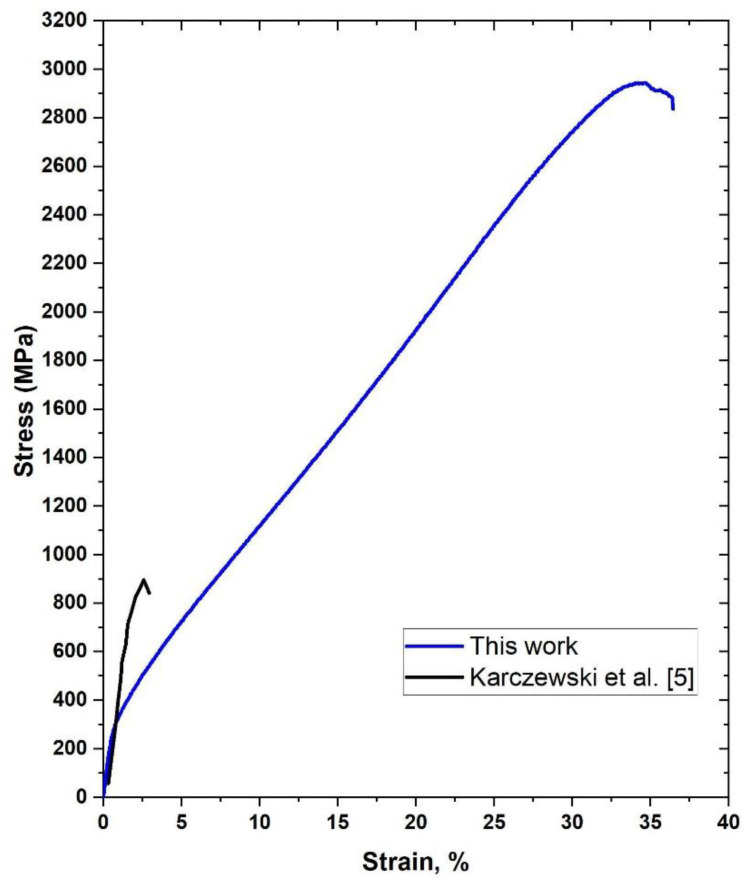
Typical stress-strain curves during the compression test at ambient temperature: black line—based on [5] and blue line—after PAIS sintering.

Because of the problems with casting FeAl alloys, powder metallurgy with the use of pure elements was found to be one of the most effective methods for cheap and effective manufacturing of these alloys. The SHS reaction occurring during the process provides an additional source of heat, improving the energetic balance of the process and usually causing porosity. The literature provides many results confirming the high brittleness of Fe–Al-based phases at room temperature [18]. Usually, the maximum deformation exhibited by these materials does not exceed a few percent [18,19]. For several years, we have been researching Fe40Al sinters obtained by pressure-assisted induction sintering (PAIS) [20,21,22,23]. Several hundred variants of sample manufacturing parameters were already made, and in all cases, sinters whose plastic properties were extremely different from those known from the literature were obtained. To show the obtained difference, an exemplary compression test course of the sample obtained by the PAIS method was applied to the literature data (Figure 2). Karczewski et al. [5] investigated the effect of temperature on the properties of the Fe40Al phase. One of the trials was the room temperature trial, which is shown in black. The blue colour is used for the stress-strain curve obtained for the PAIS sintered sample. As seen, the difference is very significant. The yield point in the PAIS sample is approximately half of that for the reference sample, while the maximum observed stress is 3-fold higher. The deformation obtained is over 30%, which is in fact an order of magnitude difference. This article aims to account for these extraordinary properties of the Fe40Al alloy obtained by the PAIS method or at least shed more light on the problem.

## 2. Materials and Methods

The principal method for fabrication of samples was reactive sintering under load in a vacuum. In these cases, iron powder (NC 100.24) was supplied by Höganäs (Höganäs, Sweden). Aluminium powder (AG 90/99.7) was supplied by Benda Lutz Company (Skawina, Poland). The exact characteristics of these powders were presented in previous works [21]. A mixture consisting of 60 atomic percent iron and 40 atomic percent aluminium was prepared. The powders were then mixed in a turbula mixer for 30 min. Mouldings (in the form of cylinders 50 mm in diameter and 9 mm in height) were then created by applying a 150 MPa uniaxial, single side load. In some studies (which were explicitly indicated), pre-alloyed powder Fe40AlZrB was used. This powder was supplied by Lermps (Belfort, France) and, in addition to iron and aluminium (40% at.), it contained zirconium (0.05% at.) and boron (50 ppm).

Raw materials were sintered using the PAIS method. The sample was placed in a graphite matrix and then pressed with graphite punches under a pressure of 50 MPa. Then, each sample was induction heated to 1000 °C and kept at this temperature for 5 min. Then, the heating was turned off, and the sample was cooled to room temperature in the chamber (typical graphs of temperature and shrinkage changes during the PAIS process are shown in Appendix A). All processes were performed in a vacuum. The chamber pressure was 3 mbar unless otherwise stated. The temperature was controlled by a thermocouple placed approx. 1 mm from the surface of the sample.

Sintered materials obtained reactively from iron and aluminium powders were subjected to homogenising annealing in air, in a muffle furnace. The homogenisation temperature was 1080 °C, and the time was 5 h. After homogenisation, the samples were cooled with a furnace. Additionally, the samples for determining the concentration of thermal vacancies were annealed for 160 h at 400 °C.

X-ray diffraction phase analysis was conducted using an Ultima IV diffractometer (Rigaku, Tokyo, Japan). A cobalt anode lamp (operated at 40 kV and 40 mA) was used to avoid fluorescence from the iron. Parallel-beam geometry was used together with a Detex Ultra linear detector (Rigaku, Tokyo, Japan). The patterns were collected in the range of 25–120° (θ/2θ) with a scanning speed of 0.5°/minute. To observe the microstructure and analysis of the chemical composition, a Quanta 3D—FEG scanning electron microscope with an EDS detector was used (FEI, Hillsboro, OR, USA). An Eclipse MA 200 (Nikon, Tokyo, Japan) optical microscope was also used to observe the microstructure. Samples for microstructural observations were prepared by grinding SiC sandpapers (600–2400) and later polishing on a cloth with diamond suspensions of 3 and 1 µm and etching by Keller’s etchant.

Microcrack analysis (for samples after compression) was performed in two ways. The first method was liquid penetrant inspection. First, the surface of the samples was cleaned with acetone. Penetrant SKL-SP1 and developer SKD-S2 (Magnaflux, Glenview, IL, USA) were then applied, and the samples were left for 10 min to soak into any cracks. After 10 min, the penetrant was removed, and a white developer was applied to the sample. After another 10 min, the samples were inspected for possible cracks. The second method was to obtain a three-dimensional image of the samples by X-ray tomography XTH 225 (Metris, Manchester, UK). The result of this study was a three-dimensional model of each sample after deformation. These models were cut with a plane parallel to the base halfway up. Half of the height was chosen because this is where the samples deformed the most, and thus, the probability of cracks was greatest.

The hardness test was conducted by applying the Vickers method with a load of 4.9 N. The hardness tests were conducted at 10–20 random places for each sample. The density and porosity were measured by applying the Archimedes method. The tension and compression tests were performed on an Instron 8501 tester equipped with a dynamic extensometer (High Wycombe, UK). The true strain of the samples was also measured by this extensometer. Compression tests were carried out on specimens with the following dimensions: diameter 4.5 mm, height 8 mm. Tensile tests were carried out in accordance with the standard EN ISO 6892-1 (Metallic materials—Tensile testing—Part 1: Method of test at room temperature).

The obtained results were analysed statistically. The arithmetic mean was calculated for all results. By contrast, as a measure of the measurement uncertainty, the standard deviation was adopted (confidence level 68%; k = 1). The *t*-test was used to compare the two results. During the *t*-test, the significance level was chosen as α = 0.05. Before performing this test, all results were checked for outliers (Grubb’s test), and the results were checked for obeying a normal distribution (Shapiro–Wilk test; α = 0.05)

## 3. Results

The obtained results shed new light on the state of the art and examples known from the literature. To enhance the reader’s experience, the research results are presented in two chapters. The first part presents the results of the research that show that the described effect of high plasticity of the FeAl phase actually occurs. In this section, we tried to prove that plastic deformation occurred by eliminating the factors that may cause a similar effect and that the findings are not the result of incorrect or over-optimistic analysis. In the second part, the possible causes of the observed phenomenon were presented.

### 3.1. Part One—An Attempt to Overthrow the Obtained Results

#### 3.1.1. Chemical and Phase Composition

As described in the methodology, the main method for producing Fe40Al was reactive sintering of iron and aluminium powders. The annealing process is very brief (only 5 min), and the obtained material is not completely homogeneous. Therefore, all samples after reaction sintering were homogenised in air for 5 h. However, if pure iron or aluminium remained in the sample or the solid solution based on iron, the sintered material could exhibit increased plasticity. Therefore, a detailed analysis of the potential presence of iron in the produced materials was performed.

**Figure 3:**
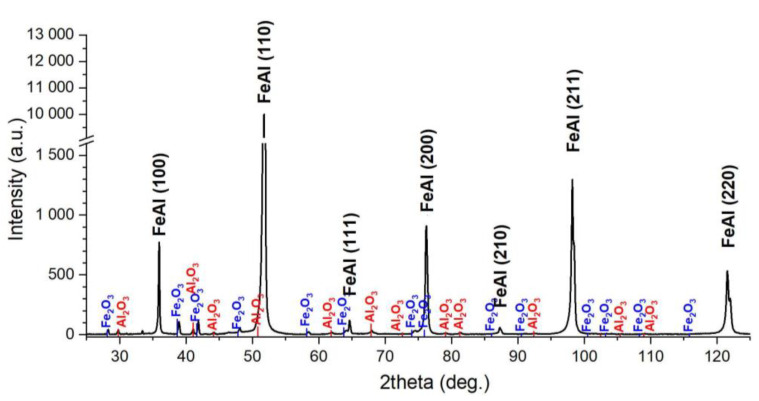
XRD analysis of the Fe40Al specimen.

The basic study was qualitative X-ray diffraction phase analysis of the samples after homogenisation, and the results are shown in Figure 3. The FeAl phase is dominating in the pattern. The presence of the peak from plane 100 (angle 36°) confirms that it is an ordered intermetallic phase and not a solid solution. Only a slight presence of iron oxides (Fe_2_O_3_) and aluminium (Al_2_O_3_) was identified. Although no quantification has been performed, the peak difference with the highest intensity indicates a trace of oxides. The intensity of the reflections from both oxide phases is approx. 1% of the intensity of the peak (110) FeAl. The small proportion of oxides is consistent with previous studies of the presence of oxides in PAIS sinters.

The XRD study showed that we were dealing with the FeAl phase. This phase occurs in a wide range of aluminium concentrations, from approximately 36 to 51%. It has also been shown that the properties of the FeAl phase strongly depend on the proportion of aluminium. To confirm that we were dealing with the Fe40Al phase, the chemical composition was tested using the EDS method. Table 1 summarises the chemical composition of five samples obtained under the same conditions. The obtained results do not differ significantly from the nominal composition. Only in the case of sample No. 4 was a slightly higher proportion of aluminium found, which should deteriorate the plastic properties rather than improve them.

**Figure 4:**
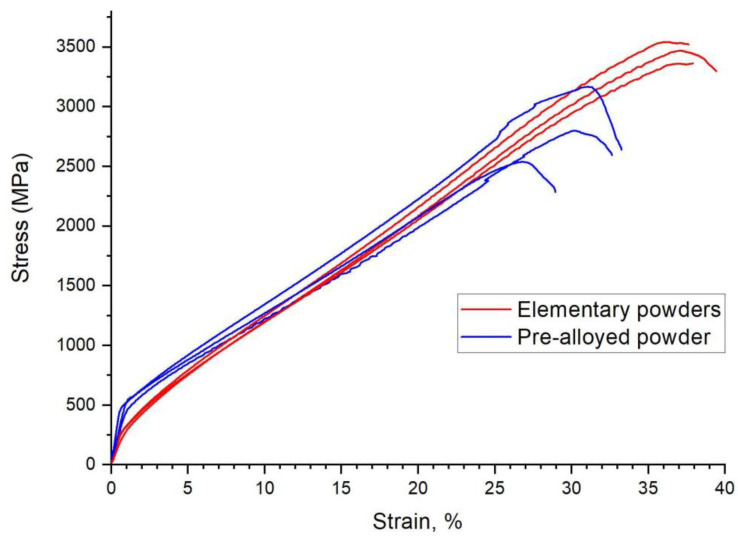
Comparison of strain-stress relationships of specimens obtained from elementary powders (**red**) and pre-alloyed powders (**blue**).

As described, the Fe40Al phase was produced in two steps: PAIS sintering followed by homogenisation. It was therefore possible that the unique properties of the finished sinters arise from homogenisation rather than sintering. To verify this idea, sintering trials of the commercial Fe40AlZrB pre-alloyed powder were conducted. Thus, the obtained sinter did not require a homogenisation process. The results of the compression test are presented in Figure 4. As you can see, the results are very similar. The sample obtained from the pre-alloyed powder was characterised by a higher yield point. At the same time, a very high (although lower than that for sintered materials made of elementary powders) compressive strength and slightly lower plastic deformation were observed (Table 2). These results mean that the high plastic properties of the sintered FeAl materials are related to the sintering method and unrelated to the material used. In addition, it has been shown that the good plastic properties cannot be caused by iron residues in the produced sinters because this iron is not present in the pre-alloyed powder.

#### 3.1.2. Morphology and Integrity of the Samples

**Figure 5:**
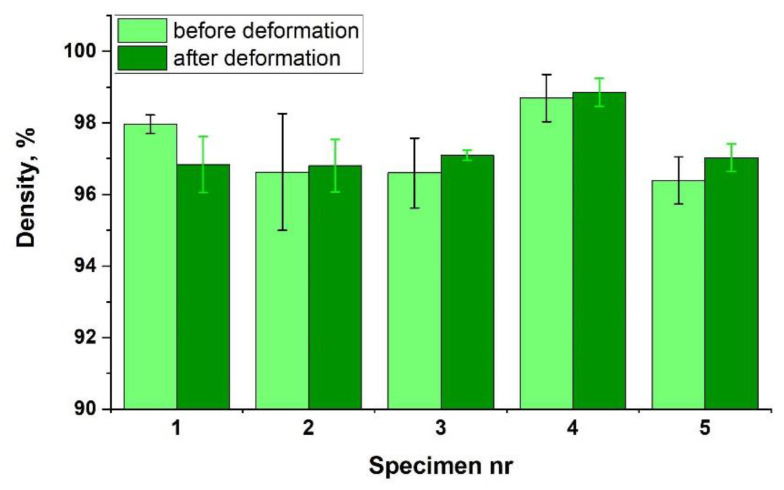
Density of specimens before and after the compression test.

Theoretically, porosity can also be one of the causes of increased plasticity. The presence of voids in the sintered material may facilitate the displacement of the grains, resulting in an increase in plasticity. To check this effect, density tests of five samples were performed. The obtained results are presented in Figure 5 (light green). The density of the obtained materials is very high and is in the range of 96–98% of the theoretical density of Fe40Al. A porosity of 2–4% cannot increase plasticity so much. However, to completely rule out this cause, an additional experiment was performed. Samples that were subjected to density tests were subjected to a compression test, and then their density was measured again. If the above-described mechanism takes place, deformation during the compression test should significantly reduce the porosity (increase the density). The obtained results are presented in Figure 5, in dark green. No significant increase in density occurred. The performed statistical test (*t*-test) showed that in all cases, the differences between the results were not statistically significant (Table 3). Thus, porosity was proven not to cause the high plasticity of the tested sinters.

**Figure 6:**
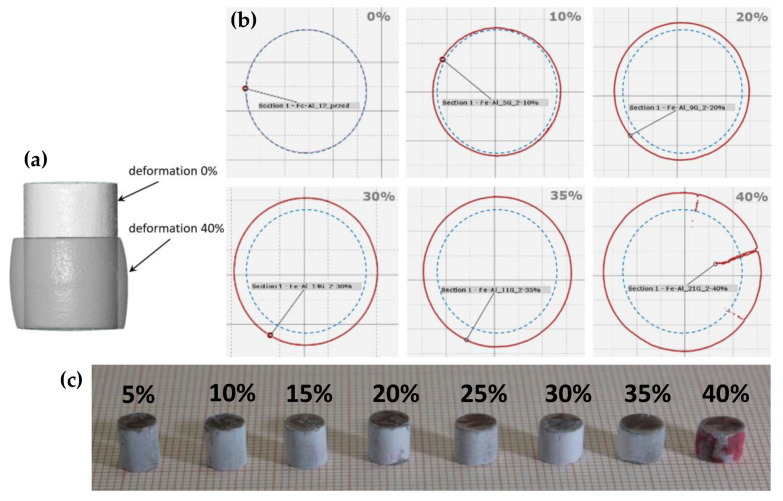
The changes in the outer contours of a sample after deformation (**a**,**b**); view of specimens after cracks detection using the penetrator method (**c**).

It was also decided to check whether the samples retained their integrity during deformation. The occurrence of cracks or microcracks along the grain boundaries may cause the grains to move relative to each other. The total effect of this displacement may be similar to plastic deformation. It was decided to exclude this effect by two methods. First, scans of deformed samples were performed using a computer tomograph. Figure 6a shows the changes in the outer contours of a sample. The sample behaves similar to a typical ductile material. As the height decreases, the diameter of the sample grows and becomes barrel-shaped. Figure 6b shows selected sections obtained at half height (the remaining sections are presented in Appendix B). The cross section of the current sample is marked in red, while the initial diameter is marked in blue, i.e., the diameter of the undeformed sample. Cracks were detected only in the sample deformed by 40%. Identical results were obtained using the second method—the penetrator method (Figure 6c). Additionally, only in this case did the specimen deformed by 40% show signs of cracking. This result confirms that the Fe40Al sinters retain their integrity until the final deformation stage.

**Figure 7:**
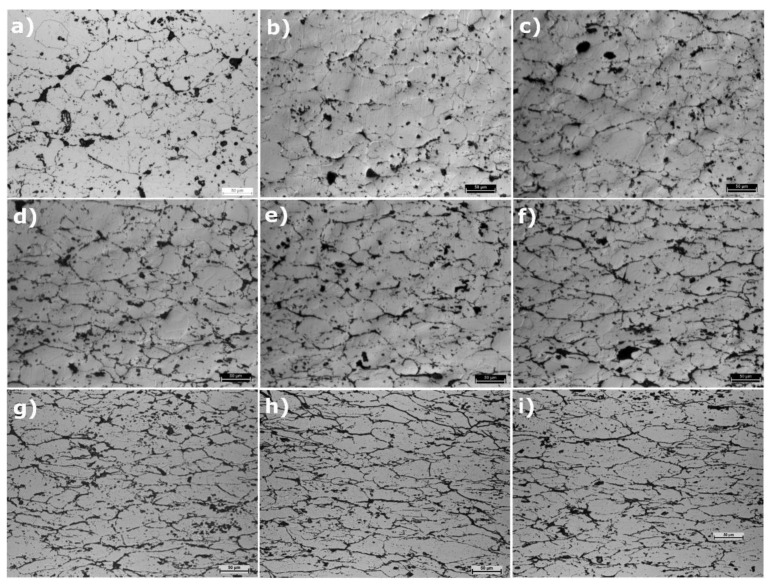
Microstructures of the specimens after deformation (in all cases, the marker length is 50 mm): (**a**) 0%, (**b**) 5%, (**c**) 10%, (**d**) 15%, (**e**) 20%, (**f**) 25%, (**g**) 30%, (**h**) 35%, and (**i**) 40%.

Observations of the microstructure (Figure 7) also confirmed the phenomenon of grain deformation in the sinters. A clear change in the shape of the grains is visible, from one closer to a circle to a more elliptical one, with increasing deformation.

All the conducted analyses of microstructure, integrity, and chemical and phase composition confirm that the material is indeed an intermetallic FeAl phase with an aluminium share of 40%. At the same time, the obtained material deforms very well at ambient temperature. It remains, therefore, to find an answer to the question of why this is so, although this material is known for its brittleness.

### 3.2. Second Part—Reasons for the High Plasticity of the Fe40Al Phase

#### 3.2.1. Comparison of the Results of Compression and Tension Tests

The results of the compression tests were very promising; however, the materials are known to have higher properties during compression than during tension. This is because, under compression, the microcracks close under the influence of compressive forces. However, when tensile, these cracks enlarge, which results in a reduction in strength. Therefore, tensile tests for sintered materials using the PAIS method were performed.

**Figure 8:**
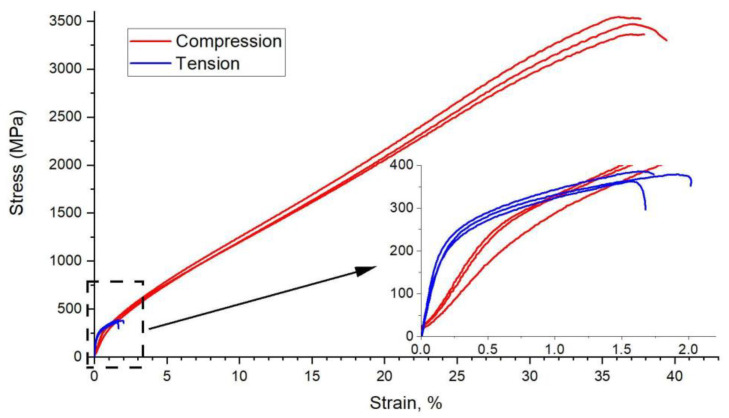
Tension test diagrams are compared with the results of compression tests.

**Figure 9:**
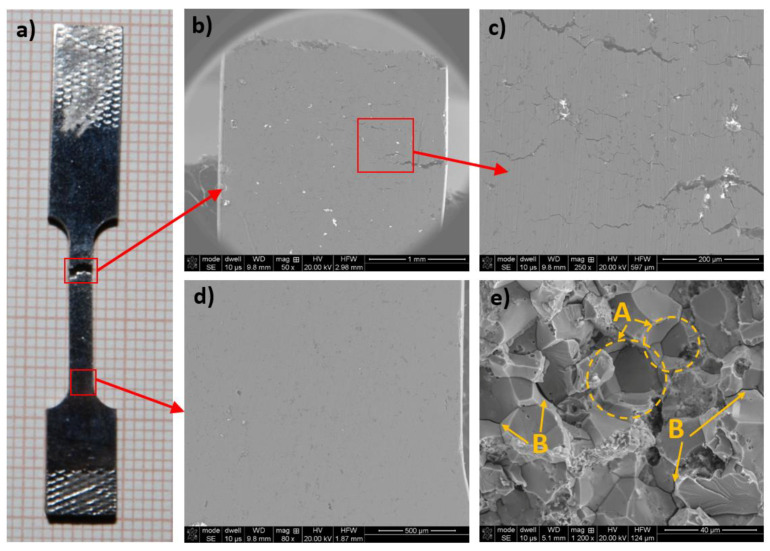
Macrostructure of the entire specimen after tension test (**a**), just below the fracture place (**b**,**c**), the area near the end of the narrowed sample area (**d**), (**e**)-the fracture view.

In Figure 8, the obtained tensile test diagrams are compared with the results of the compression tests. In Table 4, the obtained mechanical properties are compared. As you can easily see, the difference is large. When tension occurs, the samples break even at a deformation of less than 2%, which means that they behave similar to a brittle material, as is known from the literature. An almost 10-fold decrease in endurance occurs. The obtained results closely correspond to the micro- and macrostructural observations presented in Figure 9.

The apparent difference in Young’s modulus is not a real effect. During compression, samples whose height was greater than 1.5-fold the diameter were used. This value is the standard for compression specimens. In these samples, Young’s modulus cannot be correctly determined because of the presence of additional tangent stress. This stress results from the friction between the measuring table and the sample base. To eliminate this effect, samples with a height greater than 3-fold the diameter must be used. However, these samples are not suitable for determining the compression strength due to the buckling effect.

The macrostructure of the sample was observed in the two areas. Figure 9a shows the areas where the structure was observed. Figure 9b,c shows the macrostructure just below the fracture place. Figure 9d shows that the area is near the end of the narrowed sample area. Clear fractures along the zigzag line were found in the area, usually parallel to the fracture surface. This pattern of cracks suggests that cracking occurs along the grain boundaries. The increase in the length of these cracks causes them to join together, which ultimately results in the sample breaking. The number of cracks is greatest at the sample breaking point. As you move away from the fracture place, the number of cracks decreases, and practically no cracks are present at the end of the measuring area. This crack distribution is typical. Just after exceeding the yield point, the sample uniformly deforms over its entire length until it reaches the necking region. At the same time, microcracks also appear evenly along the entire length of the sample. In the area where these microcracks are the most abundant, the stresses are located, and further deformation occurs only in this area, which ultimately leads to the sample breaking in this place.

The grain boundary cracking mechanism is confirmed by the analysis of fractures after the tensile test (Figure 9e). The fractures obtained are mostly intercrystalline. The material clearly breaks along the grain boundaries, creating characteristic depressions (marked area A) after the separation of the whole grains. Additionally, gaps appear between the individual grains (B).

#### 3.2.2. Influence of Thermal Vacancies

**Figure 10:**
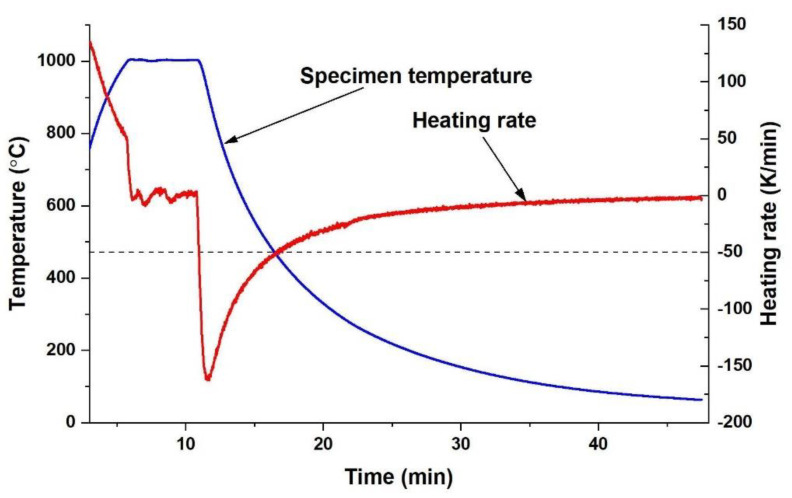
Heating rate of specimens after the PAIS process.

The literature data show that, in FeAl alloys at high temperatures, many vacancies (much more than in other metals) are formed. At the same time, the enthalpy of creating a vacancy is greater than or equal to the enthalpy of its migration. This situation means that there is a critical cooling rate. If the cooling rate is higher, thermal vacancies are not removed and remain in the material structure. The value of this critical rate is approximately 50 K/min. Figure 10 shows the temperature course during cooling of the sintered materials and the calculated heating rate. The cooling rate reaches its maximum value of approx. 150 K/min right after the start of cooling. The rate then decreases and exceeds the limit of 50 K/min when the system temperature is approximately 470 °C. At this temperature, several dozen hours are needed to remove vacancies. Therefore, the cooling rate of the PAIS process cannot eliminate thermal vacancies.

Several methods for measuring the concentration of vacancies in the material are known in the literature. This value can be determined, for example, by the Doppler broadening technique. However, the simplest and most accessible method is the hardness or microhardness measurement. In [24], the relationship between hardness and vacancy concentration was clearly demonstrated. This relationship is linear and shows that hardness increases with increasing vacancy concentration.

**Figure 11:**
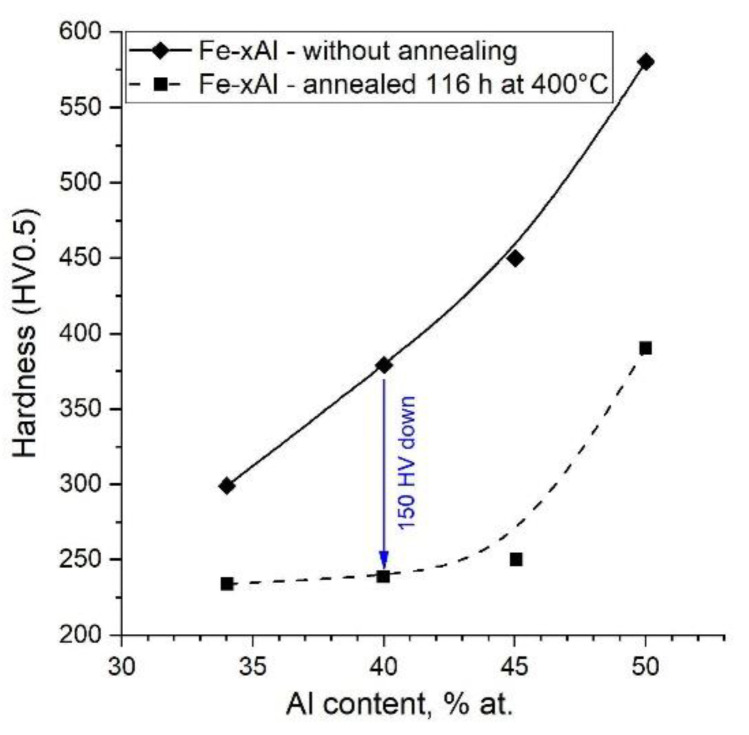
Change in microhardness of samples after annealing to remove thermal vacancies, based on [12].

**Figure 12:**
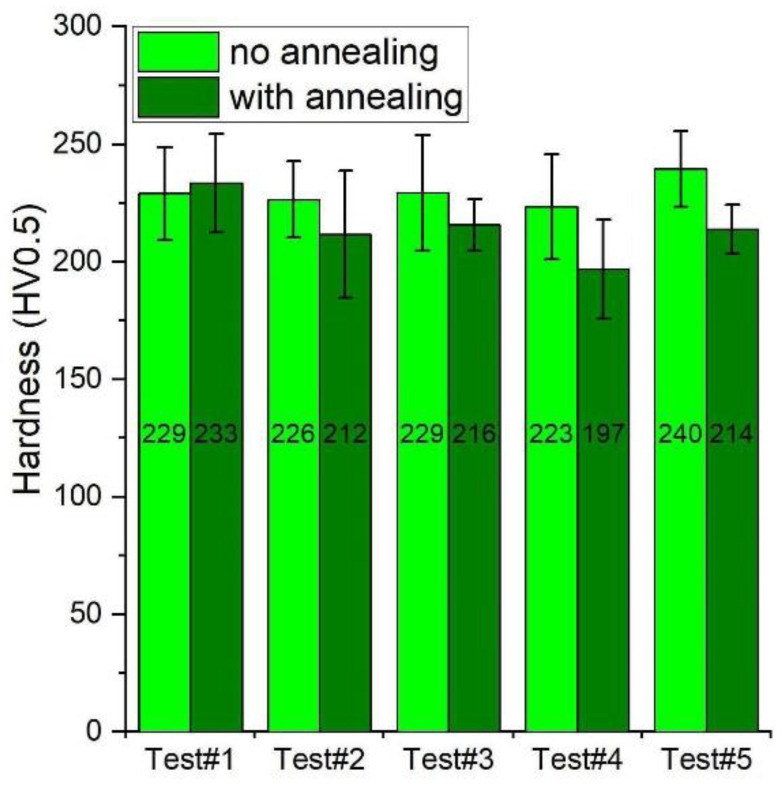
Change in microhardness after annealing to remove thermal vacancies of samples obtained by the PAIS method.

The method for eliminating thermal vacancies in the alloy is long-term (100 h or more) annealing at 400 °C. The result of this process is a significant reduction in microhardness, which is presented in Figure 11 [12]. The microhardness of Fe40Al before annealing was approx. 380 HV, while after removing the thermal vacancies, it decreased to approx. 230 HV (i.e., by 150 units). Five specimens were selected and subjected to Vickers hardness tests (HV0.5). Then, these samples were subjected to annealing at 400 °C for 160 h, and the hardness was measured again. The results are presented in Figure 12. The sintered materials without annealing have a hardness of approx. 230 HV. After long-term heating, the hardness decreases in all cases, but only slightly, by approximately 20–23 units. The statistical test (*t*-test) showed that only in the case of the last two samples was the difference in the obtained results statistically significant (Table 5). Samples 1–3 show differences within the measurement uncertainty.

The obtained results confirm that the specimens obtained by the PAIS method are characterised by a low concentration of thermal vacancies. The concentration of vacancies in the tests after sintering is practically the same as that in the samples after long-term annealing.

## 4. Discussion

The research carried out can be summarised as follows. Fe40Al sinters obtained by the PAIS method were shown to have high ductility at room temperature during the compression test. The value of the permanent deformation in the compression test is several dozen percent, which is a value unheard of in the available literature. These specimens are also characterised by a low yield point and very high compressive strength. The hardness tests showed that the reason for the good plastic properties is the low hardness of the samples immediately after sintering. What is the cause of this low hardness? All indications are that this characteristic is caused by a very low concentration of thermal vacancies. Low concentrations of vacancies in sintered materials may result from the operation of one of two mechanisms:

1. Thermal vacancies do not arise during PAIS sintering (this option seems less likely);
2. Thermal vacancies occur, but for some reason they are not frozen in the structure of the sintered materials (this explanation seems more likely).

As has been shown, the reason for the lack of vacancies cannot be a cooling rate that is much greater than the critical value of 50 K/min. However, regardless of the mechanism, the key question is, what parameter of the sintering process influences the concentration of vacancies in PAIS sintered materials?

The conducted research excluded several potential causes. One of the investigated causes was the effect of induction heating. Studies have been performed with other sintering methods that do not use induction and have shown no effect of this factor. The results of comparative studies of various sintering methods are currently being prepared for publication. After excluding other factors, it was determined that the influencing factor may be the pressure in the sintering chamber.

**Figure 13:**
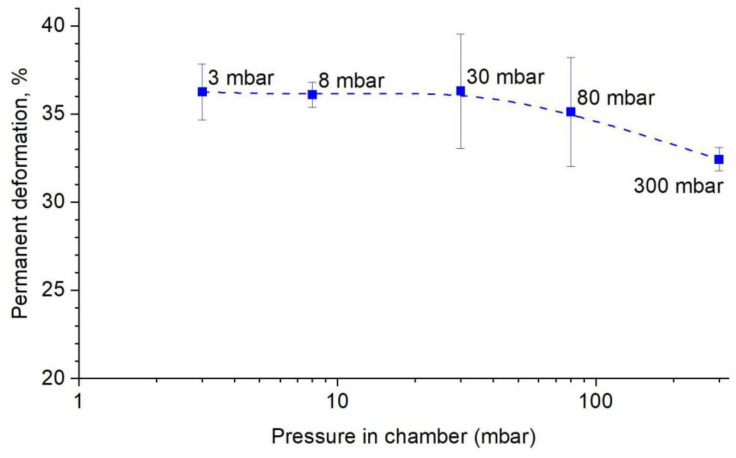
The change of permanent deformation as a function of pressure in the chamber.

Figure 13 shows the change in permanent deformation as a function of pressure in the chamber during sintering. As seen, a pressure of less than approx. 50 mbar does not affect the deformation. The samples obtained at these pressures are characterised by a deformation of approx. 36%. The increase in pressure above the limit value causes a significant decrease in deformation to the level of approx. 32%. The effect of pressure is unsurprising, as the properties of the FeAl phase are known to be negatively affected by contact with oxygen from the atmosphere. Lowering the pressure during sintering reduces the partial pressure of oxygen with which the sample comes into contact and thus reduces the “damage” caused to the FeAl phase. The novelty is that, in the available literature, it has not been reported that the reduction of contact with oxygen would allow plastic deformation of the Fe40Al phase by several dozen percent.

The question of the dramatic difference in the deformation obtained in the compression and tensile tests remains to be clarified. The mechanism of this behaviour seems simple. In classically obtained sintered materials, a large number of vacancies harden the individual grains. This hardening increases their hardness and reduces plasticity. When compressed, the material breaks similar to brittle material. In samples sintered with the PAIS method, the number of thermal vacancies inside the grains is very small, which results in low hardness and allows the grains to deform as in plastic materials (the sample takes a barrel shape). In the case of a tensile test, the failure mechanism is completely different. The absence of thermal vacancies inside the grains is of little importance during stretching because the material is quickly damaged as a result of cracking at the grain boundaries. Thus, the reason for the low tensile test properties is the low cohesive strength of the grain boundaries and not the grains as such. This mechanism confirms the nature of the fractures of the samples after stretching and the network of cracks on the surface of these samples along the grain boundaries.

In summary, it can be concluded that sintering the Fe40Al phase in a vacuum (as in the PAIS method) effectively eliminates one of the two main causes of the brittleness of this phase at room temperature. However, for this material to be widely used in industry, the second factor, i.e., low cohesive strength of grain boundaries, must be eliminated.

## 5. Conclusions

- It was proven that Fe40Al sinters obtained by the PAIS method are characterised by high plasticity during the compression test.
- Good plastic properties in this test result from the low concentration of thermal vacancies, which lowers the hardness.
- The reason for the low concentration of vacancies is the use of low oxygen partial pressure during sintering.
- The large difference in the strength obtained in the compression and tensile tests is the result of the low cohesive strength of the grain boundaries.

## Figures and Tables

**Table 1:** EDS analysis of the Fe40Al specimen.

	Sample 1	Sample 2	Sample 3	Sample 4	Sample 5
Al % at.	41.6 ± 2.2	39.3 ± 1.8	39.6 ± 1.5	43.4 ± 1.2	40.5 ± 0.6
Fe % at.	58.6 ± 2.2	60.7 ± 1.8	60.4 ± 1.5	56.6 ± 1.2	59.5 ± 0.6

**Table 2:** Strength parameters of specimens obtained from elementary powders and pre-alloyed powders.

	Yield Stress [MPa]	Ultimate Strength [MPa]	Permanent Deformation %
Elementary powders	302 ± 4	2830 ± 316	24.8 ± 0.8
Pre-alloyed powder	507 ± 42	3460 ± 90	28.8 ± 1.7

**Table 3:** Statistical comparison results (*t*-test) of specimen density before and after the compression test.

	Before Deformation		After Deformation			
Specimen nr	Density %	Stan. Dev. %	Density %	Stan. Dev. %	*p*-Value	Decision
1	98.0	0.3	96.8	0.8	0.776	negative
2	96.6	1.6	96.8	0.7	0.620	negative
3	96.6	1.0	97.1	0.1	0.839	negative
4	98.7	0.7	98.9	0.4	0.840	negative
5	96.4	0.7	97.0	0.4	0.761	negative

**Table 4:** Mechanical properties of specimens during the tension and compression tests.

	Yield Stress (MPa)	Ultimate Strength (MPa)	Permanent Deformation %
Compression	302 ± 4	2830 ± 316	24.8 ± 0.8
Tension	263 ± 8	375 ± 10	1.4 ± 0.2

**Table 5:** Results of statistical tests of the hardness of specimens with and without annealing.

	Without Annealing		With Annealing			
Specimen nr	Hardness (HV0.5)	Stan. Dev. (%)	Hardness (HV0.5)	Stan. Dev. (%)	*p*-Value	Decision
1	229	19.8	234	20.9	0.6245	negative
2	226	16.3	212	26.9	0.1389	negative
3	230	24.5	216	10.9	0.1201	negative
4	223	22.4	197	21.0	0.0138	positive
5	240	16.0	214	10.3	0.0008	positive

**Figure A1:**
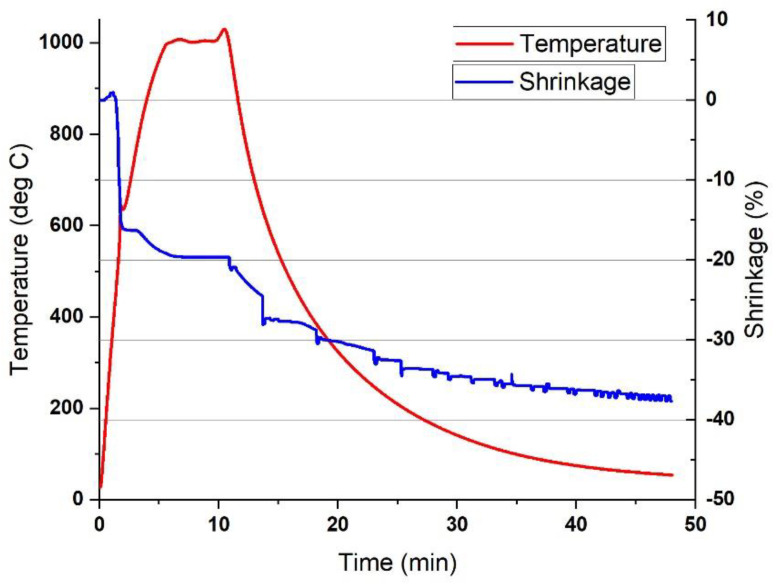
Typical course of temperature and shrinkage during sintering by the PAIS method—specimen 1.

**Figure A2:**
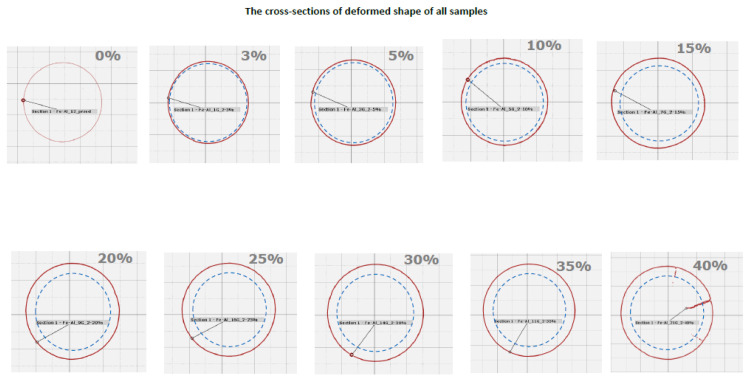
Cross section and outer contours of all samples after deformation.

**Figure A3:**
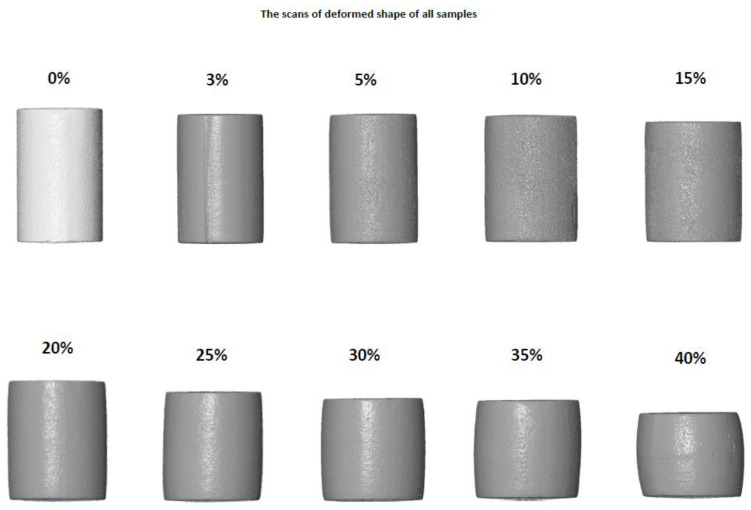
The scans of deformed shape of all samples.

## Data Availability

Data sharing is not applicable for this article.

## Appendix A

Typical graphs of temperature and shrinkage changes during the PAIS process.

## Appendix B

Cross section and outer contours of all samples after deformation.
